# Early onset sepsis calculator implementation is associated with reduced healthcare utilization and financial costs in late preterm and term newborns

**DOI:** 10.1007/s00431-019-03510-9

**Published:** 2020-01-02

**Authors:** Niek B. Achten, Douwe H. Visser, Ellen Tromp, Wim Groot, Johannes B. van Goudoever, Frans B. Plötz

**Affiliations:** 1 Department of Pediatrics, Tergooi Hospitals, Rijksstraatweg 1, 1261 AN, Blaricum, The Netherlands; 2 Amsterdam UMC University of Amsterdam, Vrije Universiteit, Department of Pediatrics, Emma Children’s Hospital, Amsterdam, Netherlands; 3 Amsterdam UMC University of Amsterdam, Vrije Universiteit, Department of Neonatology, Emma Children’s Hospital, Amsterdam, Netherlands; 4 Department of Epidemiology and Statistics, St Antonius Hospital, Nieuwegein, Netherlands; 5 Department of Health Services Research, School for Public Health and Primary Care, Faculty of Health, Medicine and Life Sciences, Maastricht University, Maastricht, Netherlands

**Keywords:** Costs, Early-onset sepsis, EOS calculator, Healthcare utilization, Newborn

## Abstract

The neonatal early onset sepsis (EOS) calculator is a novel tool for antibiotic stewardship in newborns, associated with a reduction of empiric antibiotic treatment for suspected EOS. We studied if implementation of the EOS calculator results in less healthcare utilization and lower financial costs of suspected EOS. For this, we compared two single-year cohorts of hospitalizations within 3 days after birth in a Dutch nonacademic teaching hospital, before and after implementation of the EOS calculator. All admitted newborns born at or after 35 weeks of gestation were eligible for inclusion. We analyzed data from 881 newborns pre-implementation and 827 newborns post-implementation. We found significant reductions in EOS-related laboratory tests performed and antibiotic days, associated with implementation of the EOS calculator. Mean length of hospital stay was shorter, and EOS-related financial costs were lower after implementation among term, but not among preterm newborns.

*Conclusion*: In addition to the well-known positive impact on antibiotic stewardship, implementation of the EOS calculator is also clearly associated with reductions in healthcare utilization related to suspected EOS in late preterm and term newborns and with a reduction in associated financial costs among those born term.

**Table:** 

**What is Known:** • *The early-onset sepsis (EOS) calculator is a novel tool for antibiotic stewardship in newborns, associated with a reduction in empiric antibiotic treatment for suspected EOS.*	
**What is New:** • *In newborns at risk for EOS, EOS calculator implementation is associated with a significant reduction in laboratory investigations related to suspected EOS and significantly shorter stay in those born term.* • *EOS calculator implementation in term newborns is associated with a mean reduction of €207 in costs for EOS-related care per admitted newborn.*

**What is Known:**

• *The early-onset sepsis (EOS) calculator is a novel tool for antibiotic stewardship in newborns, associated with a reduction in empiric antibiotic treatment for suspected EOS.*

**What is New:**

• *In newborns at risk for EOS, EOS calculator implementation is associated with a significant reduction in laboratory investigations related to suspected EOS and significantly shorter stay in those born term.*

• *EOS calculator implementation in term newborns is associated with a mean reduction of €207 in costs for EOS-related care per admitted newborn.*

## Introduction

The neonatal early-onset sepsis (EOS) calculator is a novel tool for antibiotic stewardship in newborns [1]. The EOS calculator estimates the EOS risk based on five maternal and four neonatal objective clinical risk factors. It stratifies newborns into three levels of risk with corresponding recommendations for management: (1) no additional care, (2) obtaining a blood culture and monitor vital signs for at least 24 hours, or (3) start treatment with empiric antibiotic therapy after obtaining a blood culture [1, 2]. This approach is associated with a reduction of empiric antibiotic treatment for suspected EOS between 41 and 45% compared with conventional strategies [2–4].

Studies evaluating the EOS calculator have provided evidence of secondary benefits associated with EOS calculator implementation, such as reductions in the number of laboratory tests and blood cultures taken [2] and the rate of admissions to neonatal intensive care [5, 6]. These findings, together with the reduction in empiric antibiotic treatment, suggest that the use of the EOS calculator may lead to a reduction in overall healthcare utilization and associated healthcare costs. This hypothesis is further supported by a recent theoretical cost-benefit analysis, which estimated a net monetary benefit of $3998 per infant with a 60% likelihood of net benefit in a US setting [7]. To our knowledge, despite signs of significant uptake [8] and multiple reports on adoption of the EOS calculator [3, 9, 10], no real-world evidence of the effect of EOS calculator use on financial costs associated with healthcare for suspected EOS has been published.

We conducted a retrospective before-after analysis in a Dutch nonacademic teaching hospital [3], to compare healthcare use and associated costs of suspected EOS before and after implementation of the EOS calculator. As we demonstrated a reduction of 44% in the empiric use of antibiotics [3], we hypothesized a significant reduction in healthcare utilization and overall financial costs in the post-implementation cohort versus the cohort before implementation.

## Methods

### Study setting, design. and patients

**Fig. 1:**
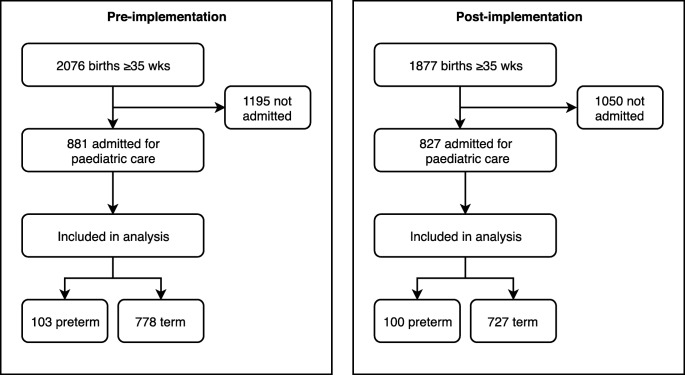
Study inclusion. Flowchart of study inclusion process

This single-center before-after EOS calculator implementation study was conducted in a Dutch nonacademic teaching hospital with a mother-child unit and a neonatal ward. The hospital provides care up to level II special care for stable or moderately ill newborns[11] and admits newborns for various reasons. Our study compared two single-year birth cohorts. We screened all newborns born in our hospital from January 1, 2014, through December 31, 2014 (pre-implementation cohort), and from April 1, 2016, through March 31, 2017 (post-implementation cohort) (Fig. 1). We evaluated all births at or after 35 weeks of gestation and included newborns admitted for pediatric care within 3 days after birth. The current study is a post hoc analysis of our implementation study, which focused on the rate of empiric antibiotic treatment in the entire birth cohort [3]. For the current analysis, we focused on admitted newborns, because it is the population susceptible to EOS care utilization and associated costs.

### Clinical practice before and after implementation of the sepsis calculator

Before implementation, newborns born at our hospital were screened for maternal risk factors and clinical symptoms by the attending staff from the mother-child unit. Maternal EOS risk factors warranting pediatric evaluation included prolonged rupture of membranes (more than 18 hours), maternal fever (38 °C or higher), prematurity, and positive maternal GBS status. Newborns requiring evaluation or care by pediatric staff for any reason were admitted for hospital care, either at the mother-child unit or neonatal ward. Newborns not admitted for hospital care accompanied mother in the mother-child unit or were discharged home.

Before implementation, a newborn at risk for EOS was assigned observation on vital signs or treatment with empiric antibiotics. This arbitrary decision was made by the attending physician, based on the combination of maternal EOS risk factors, physical examination, and/or results of complete blood count (CBC) and C-reactive protein (CRP). Within the study population, prematurity was defined as birth at 35 to 37 weeks’ gestation. If born between 35 weeks and 35 weeks and 6 days of gestation, newborns were always admitted to the neonatal ward. Without other risk factors or clinical symptoms, prematurity alone was not a reason to start empiric antibiotic treatment per se. If default empiric antibiotic therapy was started, it consisted of intravenous gentamicin and amoxicillin, followed by intravenous amoxicillin/clavulanic acid after 72 hours if not discontinued. Before the start of antibiotic treatment, blood was drawn for a CBC, CRP, and blood culture. A gentamicin serum concentration was determined and repeated if necessary. CBC and CRP were repeated after 48–72 hours of antibiotic treatment. In case of a negative blood culture after 3 days of treatment, antibiotic treatment was either stopped, or continued for clinical reasons, per discretion of the attending physician. In case of a positive blood culture, antibiotic treatment was continued for at least 7 days from start. If continued despite a negative culture, treatment was continued for 7 days.

After implementation of the EOS calculator, each birth was screened for maternal EOS risk factors and clinical symptoms, as before implementation. In case of 1 or more maternal EOS risk factors or if the newborn showed any clinical signs of EOS, prompt clinical evaluation of the newborn followed, using the EOS calculator. Based on the EOS risk calculation, in our hospital, two options were possible: either start empiric antibiotics at the neonatal ward or perform routine control of vital parameters every 3 hours at the maternal-child or neonatal ward for at least 24 hours. The EOS sepsis calculator recommendation obtaining a blood culture without starting antibiotic treatment was incongruent with our practice, and this recommendation was therefore followed by the second option. In case of antibiotic treatment, treatment protocol was equal to before implementation, as described above. Treating physicians were free to deviate from the recommendation by the calculator.

### Data collection and outcomes

Data were obtained electronically from the clinical, pharmaceutical, and financial hospital registration and billing systems. For the first outcome, EOS-related healthcare utilization, we included three groups of clinical outcomes: outcomes related to hospital length of stay, outcomes related to relevant laboratory tests (blood cultures; complete or partial blood counts; CRP; gentamicin serum concentration), and outcomes regarding (empiric) antibiotic treatment for EOS (start of antibiotic treatment, number of antibiotic days). For the second outcome, defined as financial costs related to EOS healthcare, we retrieved for each group of clinical outcomes the related costs from the hospital billing administration and calculated the combined financial cost. Costs associated with EOS-related antibiotics were calculated using costs for antibiotics in the protocol for suspected EOS described above. Because in-house billing costs were not different between the mother-child unit and the neonatal ward, these costs were not separated. To insulate our analysis from temporal cost changes during the study timeframe, we used 2017 in-house billing costs throughout analysis for both cohorts. Costs in this study represent actual cost of care, rather than final billing charges.

### Statistical analysis

Data from newborns hospitalized before implementation were compared with data from newborns hospitalized after implementation. Subgroup analyses were performed for term and preterm newborns. We also compared newborns with and without antibiotic treatment. Categorical variables were reported as (relative) frequencies with and compared with chi-square analysis. Continuous variables were reported as means with standard deviation (SD) to provide meaningful outcome measures and compared using Welch two-sample t-test, which is appropriate for skewed distributions [12–14]. All analyses were performed using R version 3.5.2 (R Foundation, Vienna, Austria).

## Results

### Inclusions

The year after implementation involved 1877 births at or after 35 weeks of gestation of which 827 (44.1%) were admitted for pediatric care in the first three days after birth, compared with 2076 births and 881 (42.4%) admissions before implementation. All admitted newborns were included in the analysis. Fifty of 827 (6.0%) admitted newborns were started on empiric antibiotics for suspected EOS after implementation, compared with 100 of 881 (11.4%) before implementation (P < 0.001). The rate of prematurity was comparable in both cohorts (12.1% after versus 11.7% before implementation, P = 0.798).

### EOS healthcare utilization

**Fig. 2:**
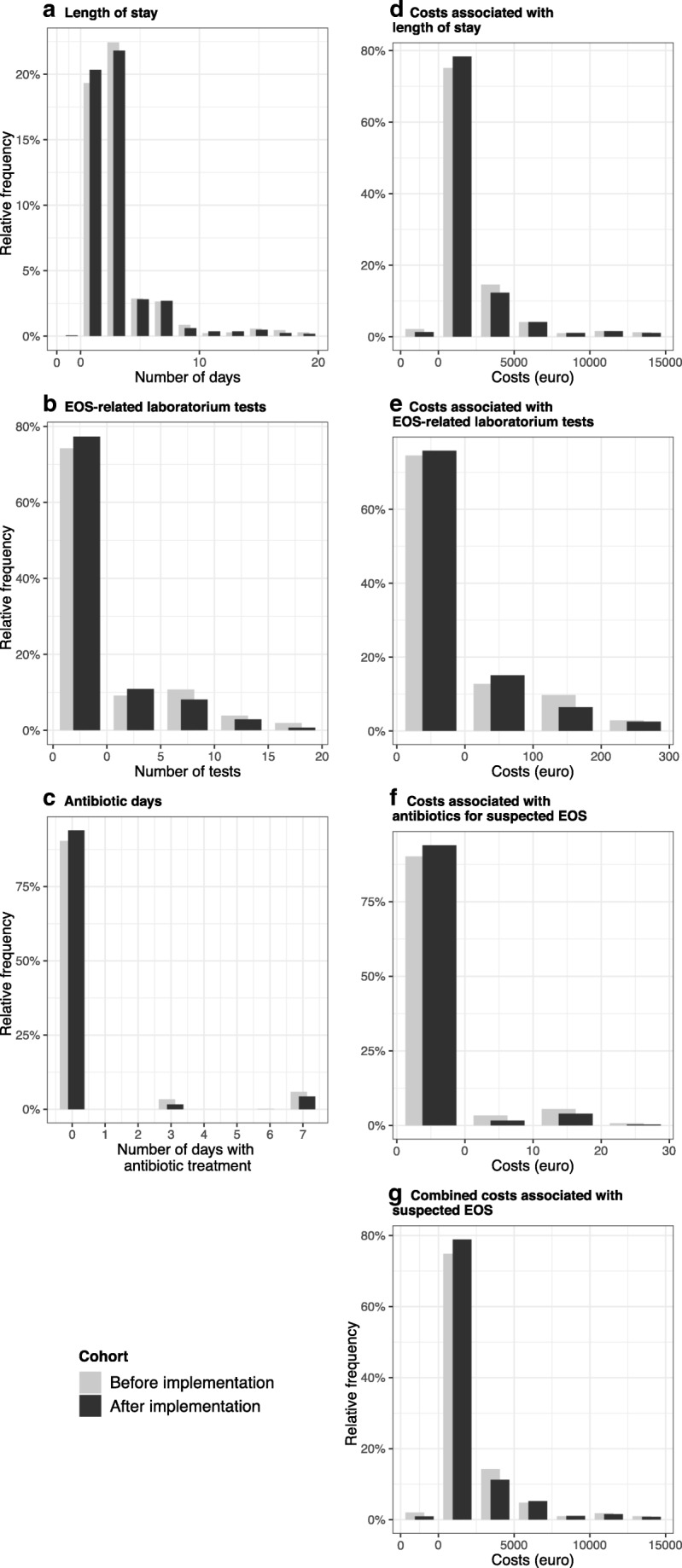
EOS-related healthcare utilization and associated costs before and after implementation of the EOS calculator. Distributions of the frequencies of clinical outcomes (panels A, B, and C) and associated costs (panels D through G), for cohorts before and after implementation. Frequency of zero as a value displayed as the first bin in continuous variables (panels **A**, **B**, and **D** through **G**). Outliers omitted from panels (**A**, *n* = 23; **B**, *n* = 9; **C**, *n* = 3; **D**, *n* = 20; **E**, *n* = 25, **F**, *n* = 1; **G**, *n* = 24) for optical clarity; no outliers were removed from analysis

Healthcare utilization was assessed for three clinical outcome groups (Table 1 and Fig. 2). Mean length of stay did not differ significantly between the two cohorts in the overall study sample but was 0.37 days shorter after implementation among term newborns specifically, (P = 0.005). We found a significant reduction in mean number of EOS-related laboratory tests per newborn after implementation (P < 0.001, Table 1), including fewer blood cultures, blood counts, CRP, and gentamicin serum concentration tests (P ≤ 0.001). The use of antibiotic treatment was significantly lower after implementation (number of antibiotic days, P = 0.009). Start of empiric antibiotics in at-risk newborns, independent of implementation, was associated with significant more EOS healthcare utilization (Table 2).

**Table 1:** EOS healthcare utilization and associated costs before and after EOS calculator implementation

	Before implementation	After implementation	P*
	N, group/N, total (%)	N, group/N, total (%)	
Overall	881 (100.0)	827 (100.0)	
Term newborns	778 (88.3)	727 (87.9)	0.798
Preterm newborns	103 (11.7)	100 (12.1)	
Healthcare utilization related to suspected EOS			
Empiric antibiotics			
Overall	100/881 (11.4)	50/827(6.0)	**<** 0.001
Term newborns	85/778 (10.9)	46/727 (6.3)	0.001
Preterm newborns	15/103 (14.6)	4/100 (4.0)	0.009
	Mean (SD)	Mean (SD)	
Length of stay in days			
Overall	3.48 (4.16)	3.27 (3.78)	0.281
Term newborns	2.95 (2.97)	2.58 (1.96)	0.005
Preterm newborns	7.48 (7.98)	8.27 (7.88)	0.475
EOS-related laboratory tests			
Overall	2.34 (4.77)	1.63 (3.62)	**<** 0.001
Term newborns	2.08 (4.28)	1.42 (3.42)	< 0.001
Preterm newborns	4.32 (7.24)	3.16 (4.57)	0.173
Antibiotic days for suspected EOS			
Overall	0.57 (1.84)	0.36 (1.47)	0.009
Term newborns	0.57 (1.85)	0.37 (1.85)	0.023
Preterm newborns	0.55 (1.77)	0.24 (1.23)	0.144
Financial costs related to suspected EOS			
Costs associated with length of stay, in €			
Overall	2614 (3034)	2516 (2737)	0.481
Term newborns	2215 (2141)	2019 (1444)	0.03
Preterm newborns	5629 (5842)	6128 (5676)	0.537
Costs associated with EOS-related laboratory tests, in €			
Overall	36.8 (89.5)	24.9 (59.2)	**<** 0.001
Term newborns	31.4 (75.6)	21.0 (54.7)	0.002
Preterm newborns	77.7 (154)	52.7 (79.8)	0.147
Cost associated with antibiotic treatment, in €			
Overall	1.54 (5.13)	0.96 (3.99)	0.008
Term newborns	1.56 (5.17)	1.00 (4.08)	0.020
Preterm newborns	1.45 (4.80)	0.64 (3.23)	0.164
Combined costs, in €			
Overall	2653 (3092)	2542 (2772)	0.434
Term newborns	2248 (2190)	2041 (1480)	0.020
Preterm newborns	5708 (5940)	6181 (5731)	0.564

*Welch two-sample t-test

**Table 2:** EOS care utilization and associated costs in at-risk newborns with or without empirical antibiotics for suspected EOS

	Treated with AB (n = 150)	Not treated with AB (n = 1558)	P*
	Mean (SD)	Mean (SD)	
Length of stay, in days	7.37 (4.88)	2.99 (3.66)	< 0.001
Number of EOS-related laboratory tests	11.2 (4.71)	1.11 (2.99)	< 0.001
No. of days with AB for suspected EOS	5.29 (2.54)	0.00 (0.00)	< 0.001
Costs associated with duration of hospital stay	5492 (3587)	2285 (2655)	< 0.001
Costs associated with EOS-related laboratory tests	194 (117)	15.3 (47.9)	< 0.001
Cost associated with antibiotic treatment for suspected EOS	14.3 (7.46)	0.00 (0.00)	< 0.001
Combined costs	5700 (3639)	2300 (2684)	< 0.001

*Welch two-sample t-test

### EOS care financial costs

Mean costs related to length of stay did not differ significantly between cohorts in the overall population but were significantly lower after implementation in the subpopulation of term newborns (Table 1 and Fig. 2). Mean costs associated with EOS-related laboratory tests and the use of empiric antibiotics were significantly lower after implementation (36.8€ vs 24.9€; P < 0.001 and 1.54€ vs 0.96€; P = 0.008, respectively). Mean combined cost associated with EOS-related care per included newborn did not differ between cohorts in the overall population but were significantly lower after implementation among term newborns specifically (2248€ vs 2041€; P = 0.020). Combined mean costs were dominated by costs related to length of stay, which accounted for 98.5% of combined costs after implementation and 99.0% before implementation.

A total of four culture-confirmed EOS cases occurred during the study period, two before and two after implementation. The mean combined costs associated with EOS-related care for these cases were €7415 per newborn. Culture-confirmed EOS represented 0.7% of total cost associated with EOS-related care in the entire study period.

## Discussion

This before-after study evaluated the effect of implementation of the EOS calculator on EOS-related healthcare utilization and the related financial costs in late preterm and term newborns. Implementation of the EOS calculator was associated with a significant reduction in laboratory investigations for suspected EOS and lower costs associated with these tests. In addition, we found that significant reductions in length of stay or overall EOS-related hospital costs associated with implementation of the EOS calculator were limited to the term newborn population.

Implementation of the EOS calculator was associated with fewer antibiotic days. Fewer newborns were started on antibiotics, but the duration of an antibiotic course was similar after implementation [3]. Therefore, observation of fewer antibiotic days is most likely due to fewer cases of “rule out sepsis” rather than fewer extended courses of antibiotics. Because each instant of blood collection and insertion of peripheral catheter for administration of antibiotics entails a painful procedure and a risk of infection, the reductions and antibiotic days and EOS-related laboratory tests imply a reduction in clinical burden and hazards. This effect may be emphasized downstream, as investigations like repeated CRP for suspected EOS lead to further investigations and longer treatment [15].

Our study shows that length of stay is the primary driver for costs in this at-risk population and that newborns treated with antibiotics have more than twofold higher EOS-related costs than those not treated (Table 2). Despite a clear reduction in antibiotic treatment in both term and preterm newborns after EOS calculator implementation, reductions in length of stay and costs after EOS calculator implementation were limited to term newborns. We suggest two explanations for the lack of clear reductions in length of stay of preterm newborns. First, the number of preterm newborns was relatively small, limiting statistical power to detect reductions in length of stay in this subgroup. Second, both prematurity in itself and related neonatal problems such as feeding difficulties warrant hospital stay, regardless of the decision to treat for EOS.

Our findings of reduced economic costs in term newborns align with a recent theoretical study by Gong et al., predicting significant costs reductions due to EOS calculator implementation [7]. For acute medical care, the model by Gong et al. predicted estimated cost savings of 1930$, equaling a relative reduction of 52%. Mean cost reduction for term newborns in our study was significantly smaller, at 207€ or a relative reduction of 9%. This may be explained by several factors. First, Gong et al. used a fictitious relative reduction of 67% in empiric antibiotic treatment by implementation of the EOS calculator, which is significantly above real-world evidence in the literature [4]. Second, the predicted cost savings were based on American healthcare costs, which are relatively high compared with European countries [16].

Finally, earlier studies reporting significant reductions in hospitalizations and other secondary benefits were performed in populations with relative high rates of neonatal ward hospitalization among well-appearing newborns and use of blood cultures without start of empiric antibiotic treatment [2]. Both of these practices are uncommon in European settings, including ours [17–19].

Strengths of this novel study include the use of robust data from electronic hospital registration systems for clinical and economical outcomes and for an unbiased determination of eligibility of patients. We included data from all admitted newborns to avoid selection bias when selecting at-risk newborns. Because the EOS calculator was applied only when a newborn was considered at-risk based on maternal risk factors or clinical symptoms, this means our results may underestimate cost reductions on the patient level associated with the EOS calculator. Although the study is inherently limited by its retrospective and temporal nature, our results are corrected for temporal cost changes, and data were available for all included newborns. Finally, our study used real-world billing costs for cost calculations, specific for our center. Different applicable costs in other centers and countries will impact the size of cost reductions associated with EOS calculator implementation.

To our knowledge, this is the first study to evaluate the effects of implementation of the EOS calculator on healthcare utilization and financial costs using non-hypothetical data from implementing the calculator in daily clinical practice. Its findings suggest that the benefits of the EOS calculator are predominantly clinical, including decreased unnecessary treatment and fewer laboratory tests. In addition, we found significant reductions in duration of hospital admission and economic costs for term newborns at risk for EOS, further reducing the burden of suspected EOS. The economic benefits will depend on healthcare tariffs and clinical protocols of a particular setting. However, the clinical benefits may very well justify implementation of the EOS calculator, even if economic benefits are modest.

## Conclusion

In addition to the well-known positive impact on antibiotic stewardship, implementation of the EOS calculator is also clearly associated with reductions in the healthcare utilization related to suspected EOS in late preterm and term newborns and with a reduction in associated financial costs among those born term.

## Abbreviations

CBC: Complete blood count
CRP: C-reactive protein
EOS: Early-onset sepsis
